# The impact of non-residential grandchild care on physical activity and sedentary behavior in people aged 50 years and over: study protocol of the Healthy Grandparenting Project

**DOI:** 10.1186/s12889-020-10024-9

**Published:** 2021-01-06

**Authors:** Marie Vermote, Tom Deliens, Benedicte Deforche, Eva D’Hondt

**Affiliations:** 1 Department of Movement and Sport Sciences, Vrije Universiteit Brussel, Pleinlaan 2, 1050 Brussels, Belgium; 2 Department of Public Health and Primary Care, Ghent University, C. Heymanslaan 10, 9000 Ghent, Belgium; 3 Research Foundation – Flanders (FWO), Egmontstraat 5, 1000 Brussels, Belgium

**Keywords:** Grandparents, Grandchildren, Caregiving, Physical activity, Sedentary behavior, Quality of life, Body composition, Prospective cohort study

## Abstract

**Background:**

Finding effective ways to support people aged > 50 years to develop adequate levels of physical activity and sedentary behavior is necessary as these behaviors are positively related to the maintenance of functional independence and health-related quality of life. Given the widespread provision of grandparental child care, examining its impact on grandparents’ energy-expenditure related behavior in the broader context of health is imperative. Therefore, the Healthy Grandparenting Project will aim to investigate the levels of physical activity and sedentary behavior, body composition and health-related quality of life in grandparents caring for their grandchildren and to compare these outcomes with non-caregiving grandparents and older adults without grandchildren, both momentarily and over time. An additional purpose is to identify possible predictors of potential changes over time.

**Methods:**

A prospective cohort study will run over a period of 2 years, including three test occasions with a one-year time interval in between (T0 = baseline, T1 = 12 months, T2 = 24 months). A total of 276 participants will be recruited in Flanders through non-probability quota sampling (50–50% men-women), of which 92 caregiving grandparents, 92 non-caregiving grandparents and 92 non-grandparents. All three subsamples will be matched for age and sex. At each test occasion, anthropometric and body composition measurements will be determined. Participants’ levels of physical activity and sedentary behavior will be assessed both objectively and subjectively by means of accelerometry and self-report questionnaires. Information about their grandchildren and the provided grandparental care (if applicable) as well as their health-related quality of life will also be assessed using self-report questionnaires. Mixed modelling will be used to identify differences in physical activity, sedentary behavior, body composition and health-related quality of life between the subsamples at baseline, as well as to evaluate and compare changes in energy-expenditure related behavior over time between subsamples and to identify predictors of the detected changes.

**Discussion:**

The Healthy Grandparenting Project is an innovative study examining the levels of physical activity and sedentary behavior in caregiving grandparents, non-caregiving grandparents and non-grandparents. Obtained results will help in the development of campaigns to maintain/improve health in adults at a more advanced age.

**Trial registration:**

clinicaltrials.gov, Identifier: NTC04307589. Registered March 2020.

## Background

European countries are experiencing significant demographic trends, including an increased life expectancy and aging society [1, 2]. As a consequence of the unprecedented growth of the older population, the number of people being a grandparent (for a prolonged period of time) is also considerable [3]. More specifically, Belgium has one of the highest prevalence rates of grandparents among people aged 50 years or older, when compared to other European countries (i.e., around 62% of men are grandfathers and 70% of women are grandmothers) [4]. Other trends in our contemporary society are the increase of female labor force participation and two-income households, higher workloads and busy social lives, as well as rising rates of divorces and single-parent or newly composed families [2, 4]. All of this leads to close intergenerational exchanges, with grandparents be(com)ing important providers of child care in addition to formal, institutional or public child care options [2, 4–7]. Especially younger (i.e., preschool-aged) children are most likely to be cared for by a grandparent without the presence of their parents [4, 5]. In Belgium, the percentage of grandparents looking after their grandchildren is 53.2% in total (i.e., 9.4% almost daily, 21.9% every week, 10.5% every month and 11.4% less often), with an average of 13.4 h of grandchild care provided in a typical week [4].

Aging, in itself, is considered a predominant risk factor for most non-communicable diseases and chronic conditions of the present time [8]. With increasing age, the incidence of high blood pressure, high levels of blood glucose, overweight and obesity expands, alongside an impairment of many physiological systems and a possible decline in functional and cognitive performance [9]. Accordingly, for middle-aged and older adults, the maintenance of functional independence and health-related quality of life is critical. Beyond the abovementioned biological changes, a more advanced age is also associated with other typical transitions into new life phases such as retirement and grandparenthood. Therefore, understanding the impact of such life-course alterations in relation to healthy aging and its social and behavioral determinants is essential [10, 11].

Given the widespread provision of grandparental child care, examining its consequences on grandparents’ health should be considered a particular focus of concern [7]. However, the existing evidence on the topic is mixed, mainly depending on the cultural or country-specific context, the intensity of grandchild care provided and the sex of the grandparent being explored [2, 6]. The larger body of (mainly U.S. based) literature suggests that co-residential and custodial grandparents experience detrimental health effects in relation to their primary caregiving responsibilities. While often coinciding with disadvantageous socio-economic circumstances, there is a growing recognition that highly intensive grandchild care can compromise both physical and mental health of the grandparent [2, 7, 12, 13]. In contrast, only a limited amount of previous research looked into grandparents’ health associated with regular or occasional caregiving (i.e., complementary to parental child care), which is far more common in today’s (European) society. This handful of studies generally found that these types of less intensive caregiving are rather beneficial for the physical and/or mental health of grandparents, and of grandmothers in particular, even after controlling for earlier life health and socio-demographic characteristics [2, 6, 7]. In addition, a study in Taiwanese older adults established reduced mobility limitations among non-residential caregiving grandparents when compared to non-caregivers [14]. Some authors already suggested that increased physical activity as a result of interacting with (younger) grandchildren, when providing such supplementary care, may have contributed to these encouraging findings [6, 14, 15]. However, research specifically investigating the relationship between grandparents’ provision of child care and (changes) in their health and health-related behaviors such as energy-expenditure related behavior is currently lacking.

There is evidence from high quality studies to strongly support the positive association between increased levels of physical activity and improved health in adults at a more advanced age [9]. Increased physical activity delays the onset and slows down the progression of functional limitations (i.e., restrictions in elementary physical and mental tasks) [16]. Previous research also indicated that physical activity reduces the risk of developing cognitive impairment [17]. Nevertheless, overall levels of physical activity are declining with increasing age and many older adults fall short of achieving the current recommendations (i.e., an accumulation of at least 150 min of moderate- to vigorous-intensity incidental or structured activities per week) [18] for health maintenance or improvement [9].

Besides physical (in)activity, sedentary behavior has emerged as a separate risk factor for health and has been identified as an independent negative predictor of successful aging in middle-aged and older adults [19]. With increasing age, the amount of time spent in (prolonged) sedentary behaviors (e.g., reading, TV viewing and computer use) has been demonstrated to expand up to 80% of waking hours [20, 21]. Yet, research has indicated that replacing sedentary behavior (even to a small extent) with light intensity physical activity results in obvious health benefits [22]. As strong independent associations of physical activity (i.e., positive relationship) and sedentary behavior (i.e., negative relationship) with overall health have been reported, older adults may benefit from the joint prescription to accumulate adequate physical activity and to avoid prolonged sitting [23–26].

The main challenge is thus to find easy and effective ways to support the middle-aged and older adult generation to develop adequate habitual levels of physical activity as well as to purposely interrupt and restrict sedentary behavior [9, 21]. Given that grandchild care usually coincides with a more advanced age and that it clearly comprises several physical tasks (e.g., lifting and carrying younger children, going for a walk, playing together, etc.) [27], it may impact on grandparents’ health indirectly through associated changes in physical activity and sedentary behavior [7, 15]. To the best of our knowledge, the role that providing care for (younger) grandchildren in a non-residential setting might play in physical activity and sedentary behavior patterns of grandparents, and by extension their body composition and health-related quality of life, is not yet examined and fully understood at this time. Therefore, the first objective of the Healthy Grandparenting Project is to provide a detailed overview of habitual levels of physical activity and sedentary behavior among caregiving grandparents in relation to their body composition and health-related quality of life. A second objective is to compare caregiving grandparents’ energy-expenditure related behaviors (i.e., physical activity and sedentary behavior) and health-related measures of interest (i.e., body composition and quality of life) with both non-caregiving grandparents and non-grandparent peers as relevant control groups. Thirdly, we want to investigate changes over time in physical activity and sedentary behavior, body composition and health-related quality of life among caregiving grandparents versus non-caregiving grandparents as well as non-grandparents both in the short and the longer term, and to identify possible predictors of these changes over time.

## Methods

### Study setting

A prospective cohort study will be implemented over a period of 2 years. Participants will include grandparents providing grandchild care in a non-residential context as well as non-caregiving grandparents and middle-aged and older adults not being a grandparent. The protocol of the Healthy Grandparenting Project and all related documents were reviewed and approved by the medical ethics committee of the local university hospital (Vrije Universiteit Brussel, Brussels, Belgium; B.U.N 1432020000017). Moreover, the Healthy Grandparenting Project will be conducted according to the ethical standards of the Helsinki Declaration and its later amendments.

### Recruitment of participants and eligibility criteria

Non-probability quota sampling will be used to recruit both male (50%) and female (50%), active (50%) and less-active (50%) adult participants at a more advanced age (i.e., 50 years and over), with a varying body mass index (BMI) and socio-economic status (SES) from convenient regions in Flanders and/or the Brussels Capital Region (Belgium), based on a pre-screening questionnaire. All three subsamples (i.e., the non-residential caregiving grandparents, the non-caregiving grandparents, and the non-grandparents) will be age- and sex-matched. Recruitment of participants will be realized through different channels including (social) media advertisement (e.g., Facebook, Twitter and popular newspapers) and contact via Belgian elderly movements (e.g., OKRA 55+; Gezinsbond). For the samples of grandparents (to be) in particular, local day care centers will also be involved to spread promotional material (i.e., posters and flyers). Furthermore, all participants from one particular subsample will be asked to provide the contact details of a counterpart from their personal acquaintances for (one or) both other subsamples central to the study, in order to facilitate the age and sex matching of the three subsamples. Additionally, the posters and flyers will also be disseminated publicly (e.g., supermarkets, libraries, cultural centers,…), in order to attract as many people as possible. A website (www.gezond50plus.be) will be set up, which will be disclosed in the promotional material, in order to create an online platform with all relevant information and news about the study, possibilities to contact the research team and the ability to sign up for participation. As selection bias might occur during participant recruitment because typically more healthy and higher educated participants volunteer for studies examining health-related behaviors [28], a collaboration with different local Public Centers for Social Welfare (CPASs) will be initiated to also recruit participants with a lower socio-economic status. Volunteering people will be requested to fill in a contact sheet including a short pre-participation screening questionnaire in order to check for the predetermined exclusion criteria: (1) being under 50 years of age, (2) not speaking the Dutch language, (3) not being able to perform independent locomotion (e.g., relying on a walking aid or wheelchair), (4) suffering from a known cognitive impairment affecting one’s memory, attention or understanding (e.g., dementia, brain injury, etc.), or (5) living in a residential care center for elderly people. More specifically, both for the caregiving and non-caregiving grandparent subsamples, the non-residential grandchild(ren) can be of any sex but need(s) to be aged between 0 and 5 years during the planned period of data collection, since providing child care is the most precarious during these early childhood years, which are characterized by limited independence of the child [6, 27]. Additionally, the pre-participation screening questionnaire will include the short form International Physical Activity Questionnaire (IPAQ – short form) [29]. People categorized as ‘low physically active’ based on the continuous IPAQ Scoring Protocol (Short Forms) will be classified as ‘less-active’ participants, whereas those categorized as ‘moderate physically active’ and ‘high physically active’ will be classified as ‘active’ participants [29]. Eligible people who are willing to participate will then be contacted by phone or email, depending on their preference, to discuss the practical arrangements regarding their study participation.

### Sample size

As to date there are no comparable studies to derive effect sizes from, a small to moderate partial η^2^of 0.02 was anticipated for the primary outcome measures (i.e., physical activity and sedentary behavior). Taking into account a significance level (α) of 0.05, a sufficient statistical power of 0.8 and an anticipated drop-out of 50% over the 2-year time course of the planned study, a minimum of 276 participants (i.e., 92 persons in each distinct subsample) is required to be recruited based on a priori sample size calculations for our longitudinal study design.

### Timing

**Fig. 1:**
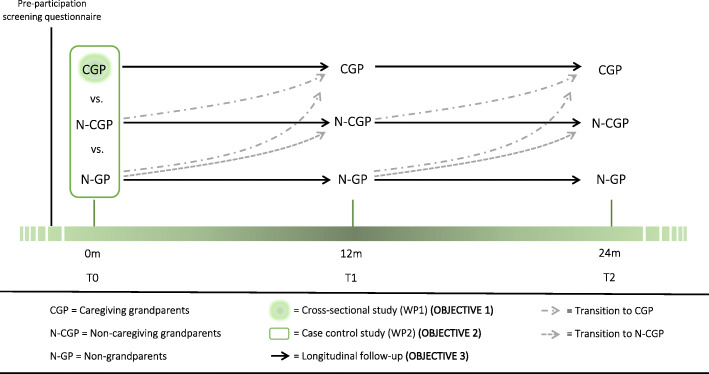
Schematic overview of the timing of data collection during the 2-year prospective cohort study

This prospective cohort study will be implemented over a period of 2 years, including three test occasions with a fixed time interval of approximately 1 year in between (T0 = baseline, T1 = 12 months, T2 = 24 months) (see Fig. 1).

### Data collection

All measurements will be performed at the respective homes of the included participants, based on a single home visit combined with the postal return of devices and data information (see Table 1). Self-report measurements of socio-demographic characteristics, intensity of grandchild care, and health-related quality of life, will be assessed by one comprehensive and integrated questionnaire. This questionnaire will be clearly explained during the home visit. Participants will be given the choice to complete this questionnaire (with its different parts) either on paper or online, using Qualtrics software in the latter case. For the self-report measurements concerning physical activity and sedentary behavior, an interview format will be used and will be completed by phone 1 week after the home visit. This interview version will be chosen as adults tend to overreport their physical activity levels with the self-administration version [30]. All objective measurements will be performed or initiated by the principal investigator (or another well-trained researcher) during the home visit.

**Table 1:** Overview of data collection measurements per test occasion (i.e., T0 to T2)

Outcome measure	Type of measurement	Instrument	Data collection approach
Socio-demographic characteristics^a^	Self-report	Questionnaire, Part 1	A
Intensity of grandchild care^b^	Self-report	Questionnaire, Part 2	A
Physical activity^c^	Self-report	Interview, Part 1: IPAQ in Dutch	C
	Accelerometer	Actigraph wGT3X-BT	B
	Activity diary	SEMA^3^: Ecological Momentary Assessment or 7-day log sheet	A
Sedentary behavior^c^	Self-report	Interview, Part 2: Sedentary behavior questionnaire for (older) adults in Dutch	C
	Accelerometer	Actigraph wGT3X-BT	B
	Sedentary diary	SEMA3: Ecological Momentary Assessment or 7-day log sheet	A
Health-related quality of life	Self-report	Questionnaire, Part 3: SF-36v2 in Dutch	A
Anthropometrics and body composition	Height	SECA 213 mobile stadiometer	D
	Weight	TANITA MC-780MA S	D
	Waist and hip circumference	Cescorf measuring tape	D
	Bio-electrical Impedance Analysis	TANITA MC-780MA S	D

A = User information provided during the home visit + Postal or online return of the questionnaire and/or log sheet

B = User information provided during the home visit + Postal return of the device

C = Information obtained by telephone interview at the end of the week

D = On-site measurement during the home visit at the start of the week

^a^All questions with regard to the demographics of grandchildren will be omitted in the non-grandparent version

^b^This part of the questionnaire will only be included for participants with one or more grandchildren aged between 0 and 5 years at the moment of testing

^c^All physical activity and sedentary behavior outcome measures will cover the same one-week period, consisting of 5 weekdays and 2 weekend days. The objective measurements require a prospective approach, whereas the self-report measurements are retrospectively questioning the same past week per test occasion

#### Socio-demographic characteristics

All necessary socio-demographic information of the participants (i.e., date of birth, sex, marital status, and possible date of retirement), and (if applicable) of their grandchild(ren) (i.e., number of grandchildren, date of birth and the sex of each individual grandchild) will be collected through a self-report questionnaire. The participants’ SES will be determined by recording their highest level of education (including the number of years of schooling) as well as their current (or prior) type of occupation and household income from salary (or pension) [31].

#### Intensity of grandchild care

Information on the intensity of the non-residential grandchild care provided in a typical week, both outside and during holidays, will also be gathered through a specific part of the integrated self-report questionnaire (only meant for the grandparents). Based on the reported intensity of grandchild care, expressed as the number of average hours per week, a distinction will be made between extensive caregivers (i.e., ≥ 30 h/week), intermediate caregivers (i.e., 10–29 h/week), sporadic caregivers (i.e., 1–9 h/week), and non-caregivers (i.e., < 1 h/week) [12].

#### Physical activity and sedentary behavior

To measure habitual levels of physical activity and sedentary behavior as the primary outcome measures, both subjective and objective measures will be included. On the one hand, self-reported physical activity will be collected using the International Physical Activity Questionnaire (IPAQ - long form, Dutch, last 7 days interview version) as a part of the telephone interview [32]. This well-known international assessment tool provides information on the frequency (i.e., number of days) and duration (i.e., in minutes/day) as well as the intensity of physical activity (i.e., light, moderate or vigorous) over the last 7 days in different domains (i.e., work-related, transportation, household and leisure-time physical activity). To subjectively measure total sedentary time and context-specific sedentary behavior, the recently developed age-specific questionnaires for assessing sedentary behavior in adults (i.e., aged: 18–65 years) and older adults (i.e., aged: 65 years and older) will also be integrated in the telephone interview [33]. This instrument is based on the Dutch version of the SIT-Q-7d [34] and assesses the time spent sitting or lying down in 11 to 12 different age-specific sedentary behavior contexts. On the other hand, wrist-worn tri-axial accelerometers (Actigraph wGT3X-BT) will be used to also objectively measure physical activity and sedentary behavior in terms of frequency, duration and intensity over that same one-week period. In order to accurately process the accelerometer data with the corresponding software (ActiLife 6), an Ecological Momentary Assessment smartphone application (SEMA^3^) will be used by the participants. A 7-day log sheet will be provided for participants not possessing a smartphone, and those preferring to complete the momentary assessment on paper. This is required to record the times (not) wearing the accelerometer, which is not water-resistant, to indicate the time of getting up and going to sleep on every single day of the one-week measurement period and to precisely indicate the type of activities performed during waking hours (i.e., including those moments when providing grandchild care, if applicable).

#### Health-related quality of life

The multipurpose 36-item Short-Form Health Survey version 2.0 (SF-36v2) will be used as an integrated part of the self-report questionnaire in order to assess participants’ well-being or so-called quality of life as a secondary outcome measure [35]. This widely used generic health status survey, originating from the Medical Outcomes Study (MOS) [36, 37], consists of eight scaled scores (i.e., physical functioning, physical role functioning, bodily pain, general health perceptions, mental health, emotional role functioning, social role functioning, vitality) yielding both a physical and mental component summary measure. Lower outcomes (e.g., a score of 0) indicate maximum or more disability, whereas higher outcomes (e.g., a score of 100) demonstrate less or no disability.

#### Anthropometrics

During every home visit per test occasion (i.e., T0, T1 and T2), participants’ anthropometric characteristics will be assessed following guidelines of the International Society for the Advancement in Kinanthropometry (ISAK) [38]. Body height (SECA 213) and weight (TANITA MC-780MA S) will be measured by means of mobile equipment allowing for accurate measurements up to the nearest 0.1 cm and 0.1 kg, respectively. From these variables, BMI (kg/m^2^) will be calculated. In addition, participants’ total and segmental body composition will be assessed using a quick and valid mobile hand-to-foot bio-electrical impedance analyzer (BIA; TANITA MC-780MA S). In order to estimate the presence of abdominal and gluteofemoral body fat, waist circumference (i.e., at the narrowest part of the waist) and hip circumferences (i.e., at the largest part of the hip) will be determined to the nearest 0.1 cm, by means of a Cescorf measuring tape.

### Outcomes

The primary aim of the Healthy Grandparenting Project is to provide an overview of the levels of energy-expenditure related behaviors (i.e., physical activity and sedentary behavior) and related changes over time among non-residential caregiving grandparents and to compare these behaviors with those of both non-caregiving grandparents and non-grandparent peers. Therefore, the primary outcome measures are participants’ levels of physical activity and sedentary behavior. Secondary outcome measures include body composition and health-related quality of life. Socio-demographic information and details about the intensity of child care (if applicable) will be included as explanatory outcome measures.

### Statistical analysis

All statistical analyses will be conducted using the most recent version of R (RStudio, www.rstudio.com). Firstly, all data will be cleaned and screened for outliers. The distributions of the (residuals of the) dependent variables will be first checked by means of Q-Q-plots. Descriptive statistics of the total sample and each of the three subsamples will be calculated. Independent samples *t*-tests and *chi*^*2*^ tests will be performed as drop-out analysis.

For all our three objectives, percentages, means and standard deviations of the levels of physical activity and sedentary behavior (i.e., primary outcome measures) as well as of body composition and health-related quality of life (i.e., secondary outcome measures) will be calculated. Mixed modelling will be used throughout the whole Healthy Grandparenting Project. For the first objective (i.e., documenting the levels of physical activity and sedentary behavior among caregiving grandparents (see green dot in Fig. 1)) the modelling will be used to examine (a) the association of the energy-expenditure behaviors of interest with participants’ body composition and health-related quality of life adjusted for the collected socio-demographic characteristics of the grandparents, as well as (b) the difference in physical activity and sedentary behavior according to the number, sex and age of the grandchild(ren) as well as the intensity of grandparents’ provision of child care. For the second objective, in which all three grandparent subsamples are of focus (see green rectangle in Fig. 1), the modelling will be used to investigate differences in both primary and secondary outcome measures between caregiving grandparents, non-caregiving grandparents and non-grandparents, while controlling for participants’ socio-demographic characteristics such as age, sex, marital status and SES. For the third and final objective of our project, the modelling will be used to evaluate changes over time in physical activity and sedentary behavior levels as well as in body composition and health-related quality of life according to the respective subsamples (see full black arrows in Fig. 1). Mixed modelling is especially well-fitted to analyze repeated measures over time as it allows to cope with frequently occurring missing values [39]. Similar analyses will be applied for those participants making a transition (i.e., transitioning to be(com)ing either a caregiving or non-caregiving grandparent for the non-grandparents and transitioning to be(com)ing a caregiving grandparent for the non-caregiving grandparents (see dashed gray arrows in Fig. 1)). All analyses will be adjusted for possible socio-demographic confounders (e.g., age, sex, marital status and SES). The mixed modelling analyses will also allow us to identify predictors of the detected changes in the abovementioned primary and secondary outcome measures.

## Discussion

Particular life-events, such as the transition to higher education or work life, changes in employment and/or in marital status as well as the transition to parenthood, all seem to affect one’s habitual daily structure [40–42]. Especially, energy-expenditure related behaviors (i.e., levels of physical activity and sedentary behavior) appear to be highly influenced by these life course transitions or alterations [40]. Becoming a grandparent is another example of such a life-changing event, which may alter middle-aged and older adults’ habitual daily structure, lifestyle and the aforementioned energy-expenditure related behaviors [43].

The Healthy Grandparenting Project is innovative as it will provide an overview of the habitual levels of physical activity and sedentary behavior in caregiving grandparents in particular (i.e., T0 – first objective). By comparing the energy-expenditure related behaviors of caregiving grandparents with those of non-caregiving grandparents and non-grandparents in a case-control design (i.e., T0 – second objective), the project will result in valuable insights into the influence of this (non-residential) grandchild care on the levels of physical activity and sedentary behavior in both women and men. The 2-year longitudinal follow-up (i.e., from T0 to T2 – third objective), in turn, will provide the opportunity to compare the changes in amount of physical activity and sedentary behavior over time within and between each of the subsamples, and additionally will make it possible to investigate the impact of possible transitions from one subsample to another (i.e., transitioning to be(com)ing either a caregiving or non-caregiving grandparent for the non-grandparents and transitioning to be(com)ing a caregiving grandparent for the non-caregiving grandparents). The new knowledge and quality evidence resulting from the Healthy Grandparenting Project will assist in a more comprehensive understanding of the effect of providing non-residential care for grandchildren on a potential increase in physical activity, decrease in sedentary behavior, as well as on body composition and health-related quality of life in an aging population. The findings resulting from our different studies will be useful for the development and evaluation of specific programs, campaigns and/or policy initiatives targeting the maintenance and/or improvement of adequate levels of physical activity and sedentary behavior and to reduce the health burden in people on the threshold of old(er) age, as well as initiatives to provide sufficient care facilities for young children (e.g., grandparental leave) in case the provision of child care by grandparents turns out to be positive.

A first strength of the Healthy Grandparenting Project is that it is the first research project to investigate the impact of providing non-residential grandchild care on levels of physical activity and sedentary behavior, as well as body composition and health-related quality of life. Earlier research focusing on grandparenthood never considered one of these energy-expenditure related behaviors as a primary outcome measure, while it is clear that both physical activity and sedentary behavior are related to health-related quality of life in both the general adult and elderly population [25, 44, 45]. The inclusion of two age and sex-matched control groups (i.e., the subsamples of non-caregiving grandparents and the non-grandparents) can be considered as a second strength of the Healthy Grandparenting Project, as this enhances the internal validity of our longitudinal study. Accordingly, a third strength of the present project is the use of a prospective cohort study design, allowing to identify predictors of changes in energy-expenditure related behaviors over time as well as to examine the effect of transitions to grandparenthood and/or to caregiving grandparenthood in both men and women. Finally, the combination of subjectively and objectively obtained measurements of physical activity and sedentary behavior as the primary outcome measures, as well as the objectively obtained body composition related data can be considered a fourth strength of this research project. Objective measurements increase accuracy and reduce measurement error, while subjective measurements provide more context-specific information on the behavior.

However, some methodological limitations also need to be considered. At first, drop-outs (i.e., from T0 up to T2) due to loss to follow-up as well as the potential loss of independent locomotion, occurrence of cognitive impairment and/or move to a residential care center for elderly people, may occur during the 2-year follow-up period. Moreover, it should be noticed that a number of participants will make a transition, with the number of non-grandparents probably undergoing the greatest decline in sample size compared to both the non-caregiving grandparents and caregiving grandparent subsamples. To be sure the Healthy Grandparenting Project ends up with sufficient participants within each of its three subsamples, enabling the appropriate statistical analyses, an anticipated maximum attrition rate of 50% has been foreseen. Furthermore, to minimize loss to follow-up, every participant will receive his/her individual test results and some personal feedback at each test occasion (i.e., T0, T1 and T2). Finally, as only Dutch speaking Flemish adults aged 50 years and over will be included to participate, the generalizability of results may be limited.

## Conclusion

To our knowledge, the Healthy Grandparenting Project is the very first prospective cohort study investigating the impact of non-residential grandchild care on levels of physical activity and sedentary behavior in people aged 50 years an older. The knowledge and insights gained from the different studies included in this project focusing on grandparenthood will help in the development and evaluation of specific programs, campaigns and/or policy initiatives aiming to reduce the health burden at a more advanced age by targeting the maintenance and/or improvement of adequate levels of physical activity and sedentary behavior.

## Data Availability

Data can be obtained from the corresponding author on reasonable request.

## Abbreviations

BMI: Body mass index
SES: Socio-economic status
CPASs: Public Centers for Social Welfare
IPAQ: International Physical Activity Questionnaire
SF-36v2: Multipurpose 36-item Short-Form Health Survey version 2.0
MOS: Medical Outcomes Study
BIA: Bio-electrical impedance analyzer
